# Neural burst codes disguised as rate codes

**DOI:** 10.1038/s41598-021-95037-z

**Published:** 2021-08-05

**Authors:** Ezekiel Williams, Alexandre Payeur, Albert Gidon, Richard Naud

**Affiliations:** 1 Department of Mathematics and Statistics, University of Ottawa, 150 Louis Pasteur, Ottawa, K1N 6N5 Canada; 2 University of Ottawa Brain and Mind Institute, Centre for Neural Dynamics, Department of Cellular and Molecular Medicine, University of Ottawa, 451 Smyth Rd., Ottawa, K1H 8M5 Canada; 3 Institute for Biology, Humboldt-Universität zu Berlin, Berlin, Germany; 4 Department of Physics, University of Ottawa, 150 Louis Pasteur, Ottawa, K1N 6N5 Canada

**Keywords:** Neural decoding, Neural encoding, Applied mathematics, Computational science

## Abstract

The burst coding hypothesis posits that the occurrence of sudden high-frequency patterns of action potentials constitutes a salient syllable of the neural code. Many neurons, however, do not produce clearly demarcated bursts, an observation invoked to rule out the pervasiveness of this coding scheme across brain areas and cell types. Here we ask how detrimental ambiguous spike patterns, those that are neither clearly bursts nor isolated spikes, are for neuronal information transfer. We addressed this question using information theory and computational simulations. By quantifying how information transmission depends on firing statistics, we found that the information transmitted is not strongly influenced by the presence of clearly demarcated modes in the interspike interval distribution, a feature often used to identify the presence of burst coding. Instead, we found that neurons having unimodal interval distributions were still able to ascribe different meanings to bursts and isolated spikes. In this regime, information transmission depends on dynamical properties of the synapses as well as the length and relative frequency of bursts. Furthermore, we found that common metrics used to quantify burstiness were unable to predict the degree with which bursts could be used to carry information. Our results provide guiding principles for the implementation of coding strategies based on spike-timing patterns, and show that even unimodal firing statistics can be consistent with a bivariate neural code.

## Introduction

The vast majority of neurons in the brain communicate complex and irregular sequences of voltage spikes—a window into neuronal information processing. These spike trains may be parsed into recurring syllables, a small set of short spike timing patterns bearing potentially different meanings. Multiple studies^1–6^ have related spike-timing patterns with different types of information. Focusing on the simplest syllables, the burst coding hypothesis separates isolated spikes from isolated bursts of spikes in rapid succession. Alternatively, bursts may merely consist of a number of equally meaningful spikes that neurons sporadically emit at a high-frequency in order to maximize information transmission^7^. Determining which coding scheme applies for a given cell type and brain region has important consequences for the interpretation of neuronal responses.

**Figure 1:**
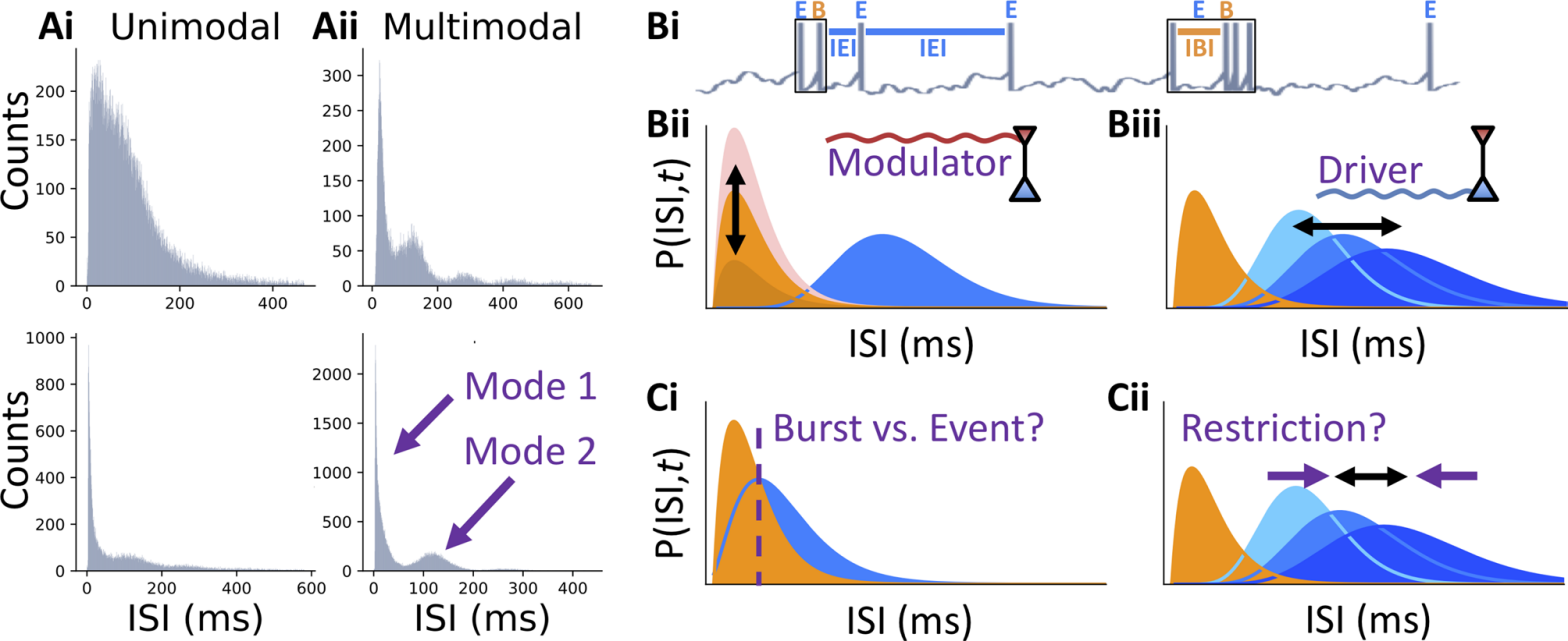
Inter-spike interval distribution and burst coding. (**A**) Experimentally recorded Inter-Spike Interval (ISI) distributions have variable shape. (**Ai**) Example ISIs from mouse visual cortex in vivo (first published in Ref.^8^), two showing a unimodal profile and (**Aii**) two showing multiple modes. (**Bi**) Burst multiplexing decomposes spike trains into bursts (black rectangles labeled with orange B) and singlet spikes on the basis of the ISI widths. We then refer to any spiking event, either burst or singlet, as an event (labeled with blue E). ISIs within a burst are referred to as Intra-Burst Intervals (IBIs), e.g. the first ISI in the second burst of the pictured spike train, while those between events are denoted Inter-Event Intervals (IEIs), e.g. 2nd and 3rd ISIs of the spike train. Observe that IBIs are usually shorter than IEIs, but not always (compare, e.g., 2nd and 5th ISIs). (**Bii**–**Biii**) Burst multiplexing assumes that the instantaneous ISI distribution, denoted as a function of time by $P({\text{ISI}}, t)$, is a mixture of two components: a burst component whose amplitude encodes the first multiplexed signal (signal 1 impinging on inset neuron) (**Bii**) and an event component whose mean encodes the second signal (signal 2 impinging on inset neuron) (**Biii**). (**Ci**) Burst multiplexing is impaired when the two components are overlapping because burst and event spikes become indistinguishable on the basis of the ISI. (**Cii**) Restricting the event component to bimodal ISI distributions may reduce the dynamic range for event rate.

A number of observations support the burst coding hypothesis. First, bursts and non-bursts occur naturally in multiple cell types and brain areas^6,9–13^. Second, bursts both cause^5,14^ and correlate with^6,15–17^ different types of information. Third, a number of cellular mechanisms regulate the generation of isolated bouts of high-frequency spikes^4^. Fourth, synaptic mechanisms route bursts and non-bursts dynamically to different targets^18,19^. Lastly, bursts have theoretical advantages for the nervous system as they allow neurons to enhance information transmission^20^, distinguish external from internal information^20^ and solve the credit assignment problem^21^.

A possible pitfall of burst coding is that many neurons do not emit well-separated singlets and bursts, thus potentially restricting burst coding to cells with a clear qualitative demarcation between these patterns. Burst coding has previously been assessed on the basis of the Inter-Spike Interval (ISI) distribution (Fig. 1A)^22–27^. A unimodal ISI distribution (Fig. 1Ai) would be expected from a neuron not using burst coding, for instance when spikes are emitted randomly at a specific rate. Alternatively, a bimodal ISI distribution (Fig. 1Aii) suggests that ISIs can be grouped into two distinct modes (Fig. 1Bi–iii): the mode on the left in Fig. 1Aii (bottom) corresponding to bursts of short ISIs, and the mode on the right corresponding to relatively well-isolated events. Does a unimodal distribution necessarily exclude a burst code? It is conceivable that neurons may tolerate a blurry separation between bursts and isolated spikes (Fig. 1Ci) if this allows them to communicate more information per spike. For instance, preserving bimodality imposes a strict constraint, which may be detrimental to information transmission (Fig. 1Cii). Thus, we may expect a trade-off between losing information to misclassified bursts/non-bursts and the cost of a restriction on the dynamic range of responses.

Here, we quantified the linearly decodable information between inputs applied to a simulated ensemble of cells utilizing the burst multiplexing^20^ burst code (see Fig. 1Bi) and readouts that mimic synaptic processing as a function of changing firing statistics. We chose burst multiplexing as our exemplar burst code for two reasons. First, it provides a tangible model of inter-cellular communication. Second, burst multiplexing is grounded in experimental results^17, 28–32^. In particular, it has been shown that inputs to different regions of a neuron’s dendritic arbor, e.g. apical versus somatic (Fig. 1Bii, iii), can be utilized to differentially drive distinct bursting, on average shorter ISI, and non-bursting, on average longer ISI, events (mixture ISI distributions in Fig. 1B,C).

Our results show that, while enforcing a bimodal ISI distribution does not appreciably affect information transmission, a burst code can still be implemented by cells with unimodal ISI histograms. Given this inability of the ISI histogram to distinguish burst-coding cell models from non-burst coders, we next asked whether other spike train statistics used for quantifying the propensity of a neuron to fire bursts of spikes^2,23,33,34^, might be equally ineffective at recognizing burst coding. We found that these metrics did not reliably distinguish burst-coding neuron models from non-burst-coding models, suggesting a disconnect between *visibly* bursty cells (e.g., with bimodal ISI distributions) and *functionally* bursty cells (i.e., those utilizing a neural code that attributes a particular meaning to bursts). Overall, our work details a rationale for using decoding approaches that separate bursts and isolated spikes even when a cell is not visibly bursty. Such approaches may reveal previously undetected streams of information within spiking data.

## Results

**Figure 2:**
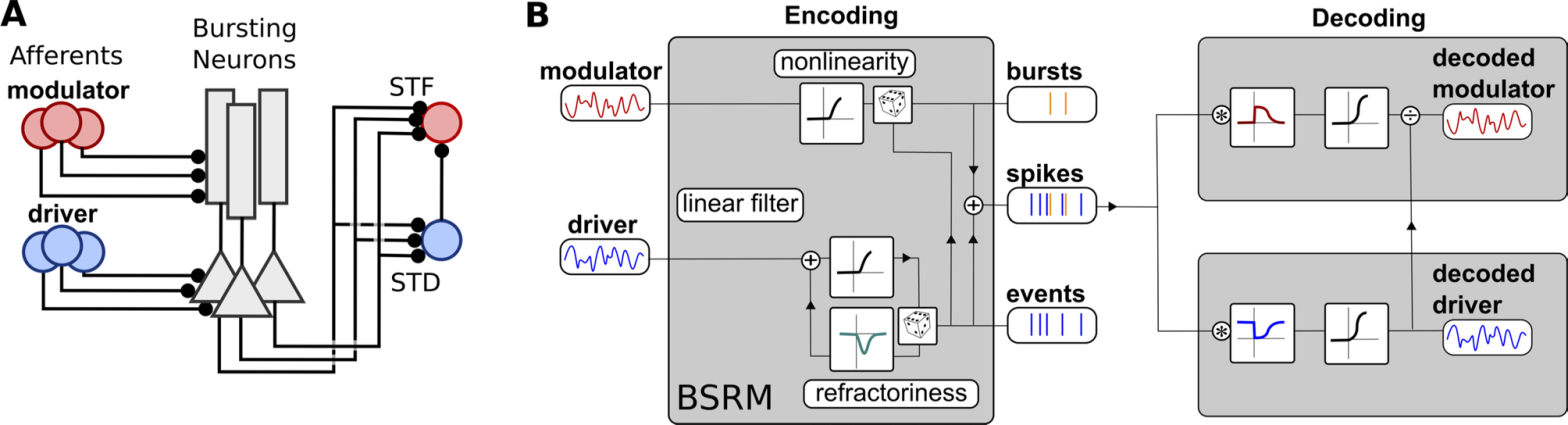
Schematic illustration of the simulation paradigm. (**A**) We model a population of bursting neurons receiving inputs from two distinct pre-synaptic populations and projecting to two post-synaptic cells with different short-term plasticity (short-term depression (STD); short-term facilitation (STF)). (**B**) Each bursting neuron is modelled with a Burst Spike Response Model (BSRM), which utilises distinct inputs to generate a bursty spike train (see supplementary Fig. S1 for sample spike trains). The driver input (blue, left) controls the event train, which is generated stochastically (illustrated by the die in the lower part) with a firing intensity that is a nonlinear readout of the input. A modulator input (red, left) controls the burst probability via another nonlinear readout. Upon event generation, a burst can be generated according to a Bernoulli process (upper die). Spike trains from this encoding cell population contain elements of both inputs. Two decoding neurons attempt to demultiplex the original signals. Different properties of short-term plasticity (illustrated by different linear filters before a nonlinear readout) extract bursts and events. A division from the event decoding cell is included to turn the estimate of the burst rate into an estimate of the burst probability, an essential step for extracting the modulator input.

We investigated the relationship between the shape of the ISI distribution and information transmission by calculating information transmission between two simulated neuronal populations while varying the properties of the network. Our modelling approach is based on a cortical microcircuit where two streams of information are impinging on two distinct compartments of a population of pyramidal cells able to produce bursts, and to route these spike timing patterns to different post-synaptic neuron populations via target-specific short-term plasticity (Fig. 2A). We considered an *encoding* population (Fig. 2B) of burst-coding cells, which received two inputs and explicitly emitted two types of *events*: single spikes and, of course, bursts. One of the inputs controlled the rate of events randomly generated amidst a relative refractory period. The other input modulated the probability that an event is a burst. When the model emitted a burst, it would add spikes to the spike train by sampling from a fixed distribution of short ISIs, the Intra-Burst Interval (IBI) distribution. Importantly, the encoding population did not force bursts to have a higher frequency than the highest achievable frequency of events. Instead, the IBI distribution could overlap with the Inter-Event Interval (IEI) distribution. We called this model the Burst-Spike-Response Model (BSRM) (see “Methods”) as it extends the spike-response model^35^. As a reference to the theory of modulator-driver inputs^36^ we called the input controlling the rate of event generation the *driver* and the input controlling the burst probability the *modulator*.

Next we considered two neurons that were post-synaptic to the encoding population and attempted to retrieve both the driver and modulator inputs without knowing which spikes were generated from which distribution. These *decoding* neurons attempted to retrieve the time-dependent signals of both the modulator and driver inputs from the output of a single, uniform population. This demultiplexing was done by allowing the two post-synaptic neurons to have different types of Short-Term Plasticity (STP), whereby the efficacy of transmission depended on the preceding ISIs. For simplicity, we modelled STP by introducing an all-or-none dependence between the amplitude of the post-synaptic potential and the previous ISI. The driver-decoding neuron was excited by a spike from the encoding population only if it was preceded by an ISI above a fixed threshold; conversely, the modulator-decoding neuron was excited only by spikes below this threshold. While real synapses do not show such all-or-none synaptic transmission, we have verified that our findings generalize to more biologically plausible models (see section “Liminal burst coding is influenced by the properties of dynamic synapses”). By previous work^20^, the driver signal was expected to be decoded by the membrane potential of the downstream cell with depressing synapses. Conversely, the neuron with facilitating synapses retrieved the burst rate, a nonlinear mixture of driver and modulator signals. To decode the modulator signal, it was necessary to take the fraction of the burst rate and the event rate, an operation that could be implemented by divisive inhibition^20,37^. Here, we calculated the quotient of the two decoding cells’ membrane potentials. Information transmission was then quantified using the linearly decodable Shannon’s mutual information rate^38–40^ (see “Methods”) by comparing the modulator and driver inputs to the membrane potential quotient and the membrane potential of the driver-decoding neuron, respectively. We refer to the information communicated between the modulator input and quotient of membrane potentials as the modulator, or burst, channel, and the analogous driver-related quantity as the driver, or event, channel.

### Potent information transmission with unimodal interspike interval distributions: liminal burst coding

**Figure 3:**
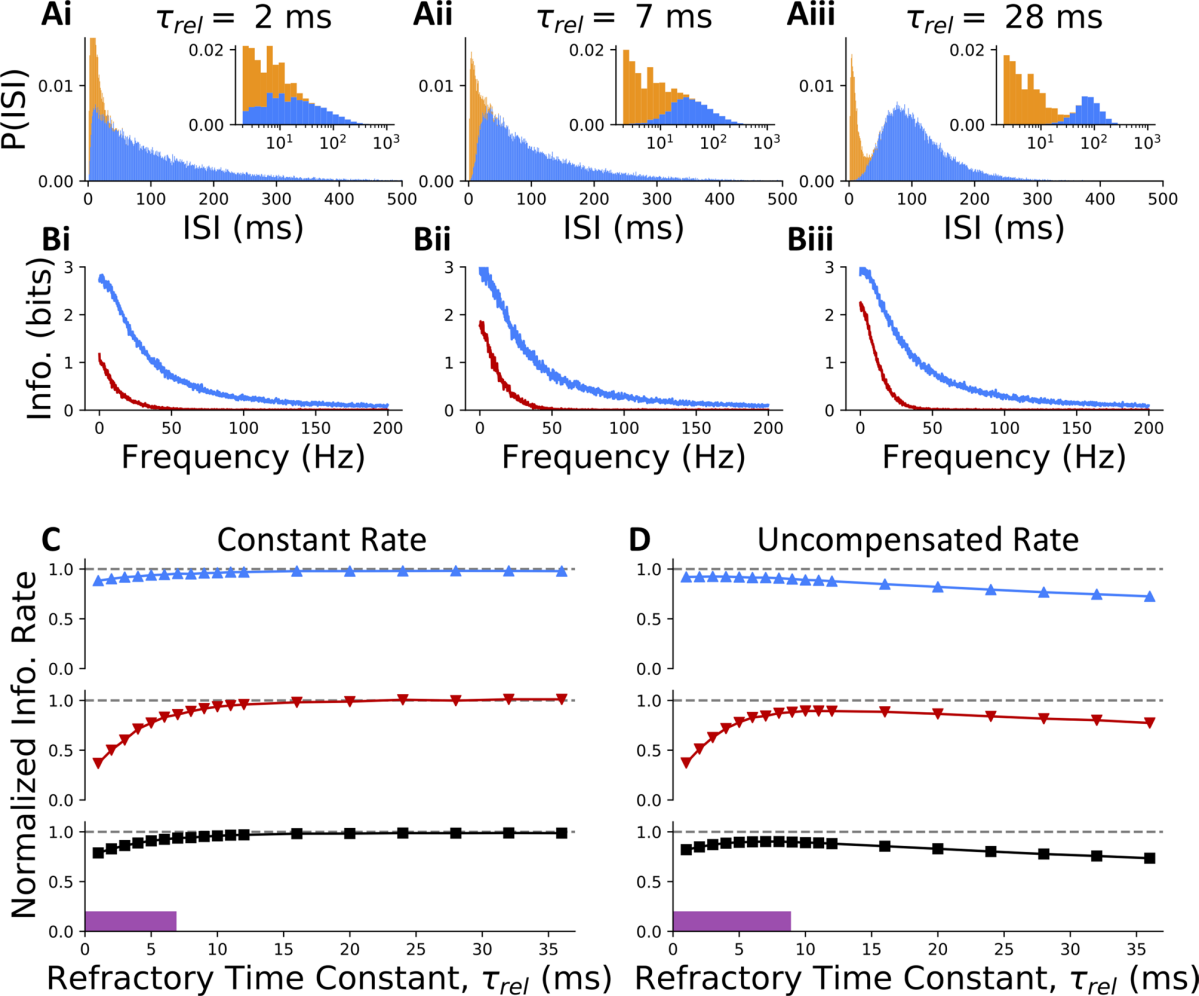
Burst coding despite unimodal inter-spike interval distributions. (**A**) The ISI distribution of the encoding population is plotted for three example relative refractory periods. Distribution is unimodal for very short $\tau _{\text{rel}} = 2$ (**i**) and short $\tau _{\text{rel}} = 7$ (**ii**) refractory periods, but bimodal for long $\tau _{\text{rel}} = 28$ (**iii**) ones (rate compensated data is shown). (**B**) A lower bound on mutual information is plotted as a function of Fourier-frequency for both the driver (blue) and modulator (red) channels. Integrating the lower-bounded information over frequency and summing driver and modulator channels produces estimates of linearly encoded information of (**i**) 131.58 bits/s (**ii**) 148.91 bits/s, and (**iii**) 156.64 bits/s for each distribution shown in (**A**), respectively. (**C**) Normalized Information rate is shown as a function of relative refractory period for the driver channel (blue, up-arrowhead), the modulator channel (red, down-arrowhead) and both channels together (black, square). The purple bar indicates the region of parameter space producing visually unimodal ISI distributions (those exhibiting only a single peak). For each value of the relative refractory period, the firing threshold for neurons in the encoding population was scaled to preserve the same average firing rate for all values of the relative refractory period. (**D**) As in (**C**) but without adjusting cell firing threshold. Normalization in (**C**,**D**) is by the maximum (over $\tau _{\text{rel}}$) linearly decoded information rate, for perfectly decoded events and bursts.

**Figure 4:**
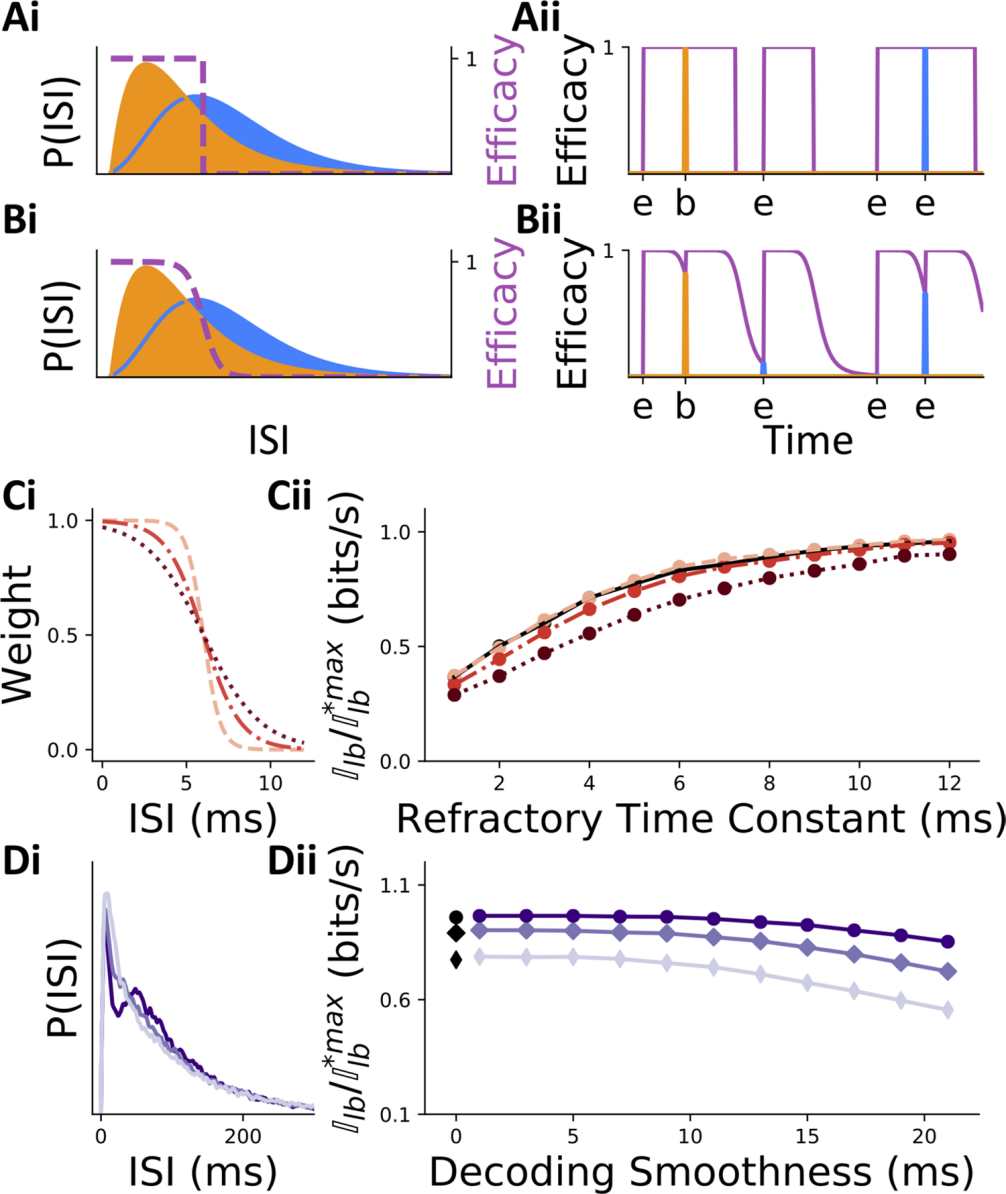
Properties of dynamic synapses influence information transmission. (**A, B**) Bursts and events can be decoded by either sharp (**A**) or smooth (**B**) dependence of the synaptic efficacy on the interspike interval. (**Ai–Bi**) IBI (orange) and IEI (blue) distributions are overlayed on the function relating interspike intervals to synaptic efficacy (purple, right axis). (**Aii–Bii**) The effect of this function on the decoded modulator signal is illustrated using a single hypothetical spike train with true events (ticks labeled by ‘e’) and true intra-burst spikes (ticks labeled by ‘b’). The strength at which an incoming spike will be read out is represented by the height of the blue and orange vertical lines (event = blue; intraburst = orange), whose heights are themselves decided by the momentary synaptic efficacy (purple line), which changes in time as a function of spike history. (**Ci**) Three instances of graded sensitivity to the firing frequency. (**Cii**) Modulator information transmission is shown against the refractory time constants around the liminal regime for the three nonlinear sensitivity functions shown in Bi (same colour and line-style schemes—rate compensated data shown). Black line (obscured behind yellow) gives sharp threshold-based communication for comparison. (**Di**) ISI distributions (smoothed with Gaussian filter) for three different relative refractory period time constants: 5 ms (light purple), 8 ms (purple), 12 ms (dark purple). (**Dii**) Modulator information transmission as a function of the sensitivity to firing frequency for the three ISI distributions pictured in (**iii**) (same colour scheme). Black dots denote sharp threshold-based communication. All data for variable $\tau _{\text{rel}}$ is rate corrected, as in Fig. 3C, and data for sharp threshold communication is taken from Fig. 3.

To manipulate the shape of the ISI distribution, we varied the parameter for the relative refractory period of events, $\tau _{\text{rel}}$. Small to medium values of the relative refractory period allowed very small IEIs such that the IEI and the IBI distributions overlapped (Fig. 3Ai–ii). For short relative refractory periods, the overall ISI distribution was unimodal and the resulting spike trains had the appearance of a Poisson process (i.e. an exponential distribution). Large values of the relative refractory period were associated with events that were further apart (Fig.3Aiii). Since these could be either singlets or bursts, this larger relative refractory period resulted in sporadic bursts of spikes, a regime that should be ideal for burst multiplexing^20^. In Figs. 3 and 4, we modelled bursts as consisting of two spikes only, both for modelling simplicity and because research has observed doublet bursts occurring more frequently than longer bursts^9^. The consequence of this simplifying assumption will be investigated later in this article. When assessing how changing the relative refractory period affected information transmission, we considered two conditions and two decoders. In the *constant rate* condition, the cell firing threshold was adjusted to compensate for the changes in firing rate incurred by modifying the relative refractory period. Alternatively, we also simulated responses where we only varied the relative refractory period and allowed the firing rate to change accordingly. This setup will be referred to as the *uncompensated* condition. The first of the two decoders we employed used a model of synaptic plasticity to decipher events from bursts. Since information loss is likely to arise from a misclassification of burst and singlets, the second decoder considered was a *perfect decoder*. The perfect decoder did not rely on the ISI for burst identification but was given the information of whether a spike was generated by the driver or modulator input. Together, these different conditions and decoding approaches allowed us to determine how burst coding depends on the bimodality of the ISI distribution.

As a first step toward estimated information transmission, we calculated the linearly-decoded information spectrum between the driver signal and driver-deocding neuron. This quantity showed high information for low input frequencies, which slowly dropped to zero for very rapidly-changing inputs (Fig. 3B-blue lines). For the modulator signal, a low-pass profile is also observed, but with generally less information and a lower cutoff frequency (Fig. 3B-red lines). These observations are exactly what is expected from the theoretical properties of the ensemble-burst code^20^, whereby burst coding is optimal for the communication of more slowly changing inputs. In addition, the low stationary burst probability (here $\approx$ 0.2, chosen to match observations in cortex^9,17^) is expected to reduce the proportion of information transmitted by bursts. To arrive at a more global measure of information transmission efficacy, we integrated the information spectrum over the linearly decoded information at each frequency^40^, resulting in a measure of information rate in bits/s.

To arrive at a measure of the total burst multiplexing information, we calculated the sum of the information rates of the driver and modulator channels (see “Methods”). In the constant rate condition, we found that both the driver and modulator channels were best transmitted as the ISI distribution of the encoding population became more bimodal. In this limit, both channels matched the perfect decoder (dashed lines in Fig. 3C, D). The driver channel was almost unaffected by the changing ISI interval distribution, transmitting 90.13% of the information even when almost the entire IEI distribution overlaped with the the IBI distribution (see Fig. 3Ai), with a 2 ms relative refractory period for events. Showing a drop to 50.10% in information at $\tau _{\text{rel}}=2$ ms, the modulator channel was more profoundly affected by using a short refractory time constant. Information started to degrade weakly just before the transition from bimodal to unimodal ISI distributions (at a relative refractory period of 7 ms), but the impediment remained weak when the ISI distribution became unimodal (purple bar in Fig. 3C). A stronger transition to low information transmission occurred at a relative refractory period of 5 ms. As the driver contributes more information than the modulator channel, the total burst multiplexing information observed only a moderate decrease in information transmission (Fig. 3C black), and this at the highest overlap between distribution modes. We refer to the range where ISI distribution is unimodal but the IEI and IBI show minimal overlap as the *liminal* regime. In this regime the IBI and IEI distribution join but hardly overlap, and thus bursts/non-burst can be separated reliably. We also note that an important fraction of modulator information (40–60% of perfect decoder) could be transmitted even when the IBI and IEI distribution entirely overlapped. Together, this highlights the possibility for cells without a clear demarcation between bursts and single spikes to use burst coding almost as effectively as cells with perfectly separated firing patterns.

In the uncompensated condition (Fig. 3D), both the driver and modulator information were decreased with increasing bimodality. This reflects the effect of decreasing firing rate caused by increasing relative refractory periods, which naturally affects the information transmission. As a consequence, this condition is associated with a maximum of the modulator information over the the range of relative refractory period tested, a maximum that occurs in the liminal regime.

### Liminal burst coding is influenced by the properties of dynamic synapses

Having established the possibility of burst coding in unimodal regimes (Fig. 3), we investigated the robustness of this result to changes in our model parameters. First, we revisited assumptions made on the properties of synaptic dynamics enacting the decoding. In particular, we were interested to know whether liminal burst coding could also take place when synaptic transmission was a smooth function of firing frequency—as observed in experiments—instead of the all-or-none dependence assumed in the previous section. To use a more accurate model, we have simulated synapses whose dynamics follow a linear-nonlinear cascade, which was recently shown to provide an accurate description of STP at the mossy fiber synapse^41^. In this model, the sensitivity to firing frequency is controlled by a convolution kernel, which we refer to here as the sensitivity function. Real neurons have smooth sensitivity functions which are well captured by either a multi-exponential decay or a mixture of Gaussians^41^. Using a sigmoidal function, we could interpolate between a sharp, (Fig. 4Ai–ii) and a more realistic, smooth (Fig. 4Bi–ii) dependence of synaptic efficacy on the previous interspike interval. We hypothesized that a more graded dependency would perturb burst coding, as each variation in ISI would be transmitted as a fluctuation in post-synaptic potential amplitude. Our question was whether this perturbation would render burst coding ineffective or could be viewed as negligible.

Figure 4C shows results for the modulator signal only (see supplementary Fig. S2 for driver channel), as it is more strongly corrupted by unimodal ISI statistics, although the sensitivity to firing frequency was modulated in both burst and event channels. Decreasing the sensitivity of the decoding synapses to firing frequencies (i.e. increasing the smoothness) made the drop in information due to bimodality more graded and tended to reduce burst coding efficacy (Fig. 4C,D). These results demonstrate that burst transmission is reduced slightly when synapses have graded sensitivity to firing frequency; however, this reduction is negligible when the graded sensitivities are appropriately steep.

### Burst length and low event rates

**Figure 5:**
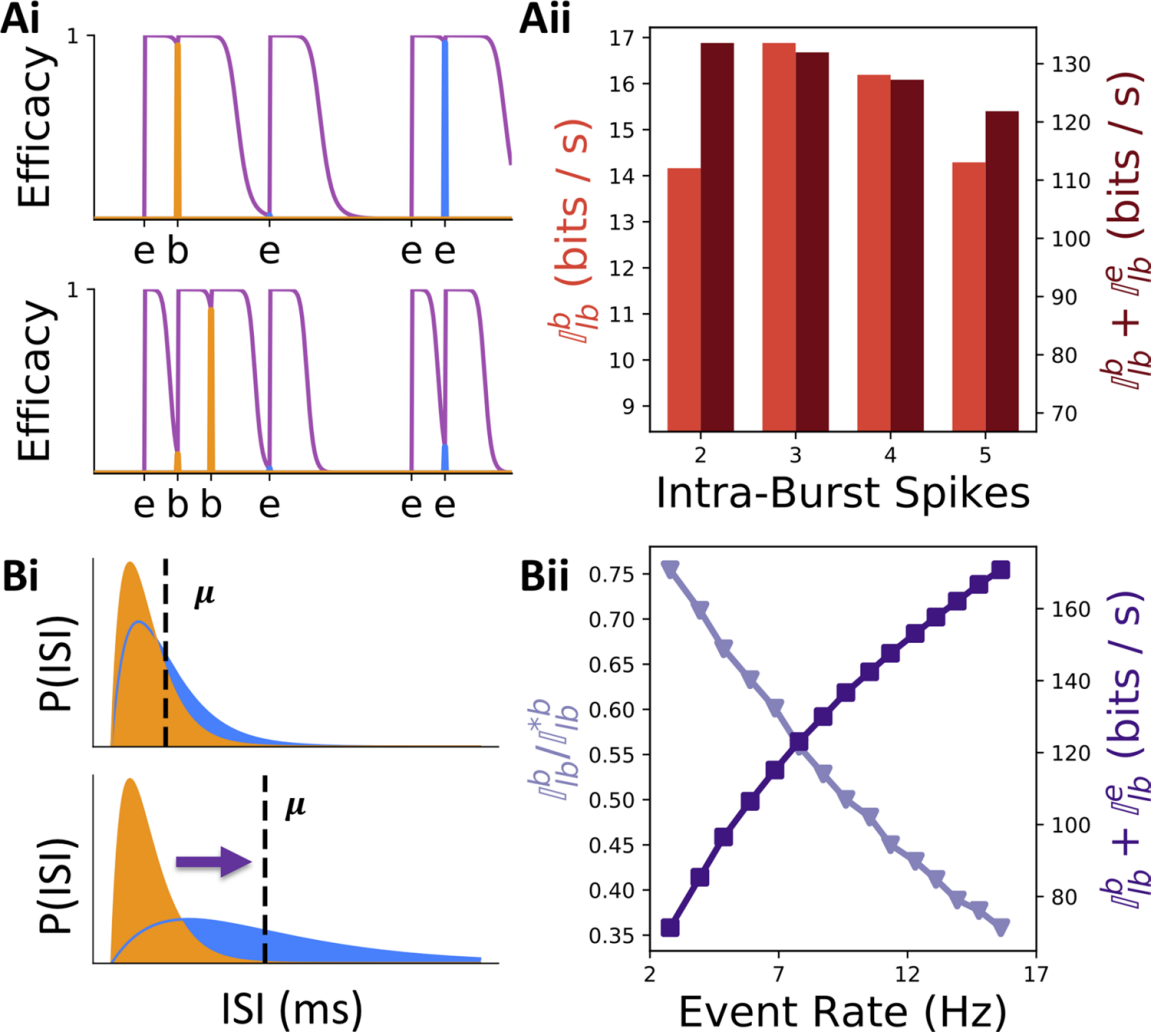
Effects of burst length and event rate on information transmission. (**Ai**) Illustration of the modulator-channel communication of a single hypothetical spike train made of a number of true events (‘e’ ticks) and bursts (‘b’ ticks) with IBIs comparable to IEIs (as in Fig. 4Aii, Bii). Top: for bursts made of 2 IB spikes, to communicate the burst implies that an event with a similar interval (last event) will also be communicated. Bottom: using bursts made of 3 IB spikes (orange) allows the burst to be transmitted while minimizing transmission from the last event because the synaptic efficacy is allowed to accumulate through successive IBIs. (**Aii**) Modulator channel information rate (left y-axis) and total information rate (right y-axis) are shown as a function of the number of intra-burst spikes. (**Bi**) Schematic illustration of how the overlap between a fixed IBI distribution (orange) and the IEI distribution depends on whether the average IEI ($\mu$, dashed line) is short (top) or long (bottom). **Bii** Normalized burst channel decoding efficacy (left y-axis, light purple down-arrowheads) monotonically decreases, with increasing average event rate, while the total burst multiplexing information (right y-axis, dark purple squares) increases. Both Aii and Bii were generated using strongly unimodal ISI distributions ($\tau _{\text{rel}} = 2$ ms).

To further interrogate the robustness of burst coding in the unimodal ISI distribution regime, we looked at two other parameters that contribute to the efficacy of burst coding. The first parameter we investigated was burst length. We therefore varied the number of spikes following the first spike in a burst, namely the number of intra-burst (IB) spikes. The burst multiplexing code used here differentiates only between bursts and singlets, meaning the precise number of intra-burst spikes does not carry information. However, changing the number of spikes can affect burst coding fidelity: if burst length is increased from doublets to longer bursts of *N* IB spikes, the STP rule of the decoding cell can be adjusted (see “Methods” section) to respond only to spikes after being primed by $N - 1$ IBIs (Fig. 5Ai). Such a dependence between the number of intra-burst spikes and the changes in synaptic efficacy is observed in experiments^41^. Since the probability of observing *N* subsequent events with short ISIs decreases as *N* increases this allows post-synaptic cells to better distinguish events from bursts, regardless of the overlap between IEI and IBI interval distributions.

We found that increasing burst length increased burst channel information, particularly for 3–4 IB spikes (Fig. 5Aii). The total information, however, always decreased with increasing burst lengths. We know of two factors limiting information transmission as burst length is increasing. First, there is a poorer temporal alignment between the burst generating signal and the transmission of bursts due to an increased number of randomly sized intra-burst ISIs between burst generation and burst detection. This error could, in theory, be reduced in cells with less randomness in their IBIs. Second, though they improve burst transmission, longer bursts do not contribute any new information to a spike train but still occupy significant periods of time, causing fewer event spikes to be fired per unit time and thus decreasing information transmission rate for events. The latter effect was discussed by Naud and Sprekeler^20^, where it was shown that increasing burst length is detrimental in general, but that study did not take into account the influence of synaptic properties on total information transmission. Our results supplement this theoretical study and indicate that increasing the length of bursts to more than two spikes per burst is beneficial only to the communication of a modulator signal.

Next we considered reducing the average event rate. In Fig. 3C, we had kept the average event rate fixed to a value close to 10 Hz. Reducing the average event rate results in a lower spike rate and thus lower information rate, but also reduces overlap between IEI and IBI distributions (Fig. 5Bi), thus limiting spike misclassification error and improving burst channel efficacy. To quantify the relative improvement in burst-decoding as event rate is decreased, we performed simulations using clearly unimodal ISI distributions ($\tau _{\text{rel}}=2$ ms) and varied the average IEI. To quantify information transmission amidst changing firing rate, we normalized the constant-rate information transmission, ${\mathbb {I}}^b_{lb}$, by the information calculated with perfect decoding ${\mathbb {I}}^{*b}_{lb}$. For the transmission of modulator information, we found that transmission improves as event rate decreases (Fig. 5Bii-light purple). Concomitantly, the total information rate decreases with decreasing event rate (Fig. 5Bii-dark purple). Together, we found that the average event rate controls a compromise between the communication of the modulator input and the communication of the net amount of information even when IBI and IEI distributions overlapped.

### Classic methods fail to identify functional burstiness

A large body of research has been devoted to distinguishing cells that burst from non-bursty cells^12,42–44^. Some of the methods developed to this end^23,26,33,34^ have focused on classifying cells based on the similarity of their response statistics to those of the Poisson process. Other approaches have focused on the identification of separate peaks in the ISI distribution or the auto-correlation function. Given that a population of neurons can communicate burst-coded information efficiently despite a unimodal ISI distribution, we now ask whether this disconnect between the bimodality of the ISI distribution and the functional role of bursts extends to other metrics for classifying bursty cells.

**Figure 6:**
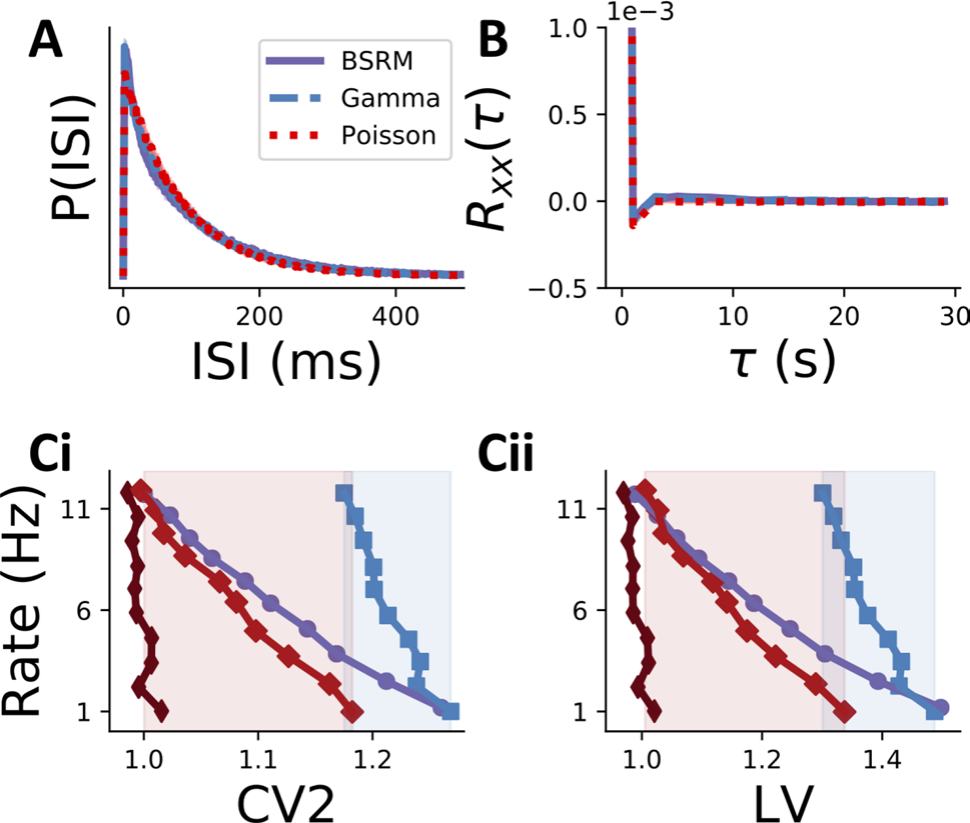
Common metrics of spike train irregularity do not distinguish functionally bursty cells. (**A**) ISI distribution of the BSRM model in effective multiplexing regime (solid, purple) is almost identical to that of a homogeneous, refractory, gamma renewal process (dashed, blue), and highly similar to that of a Poisson process (dashed, red). (**B**) Autocorrelation of spike trains from (**A**) are almost identical. Y-axis units are [spikes]^2^. (**C**) Plotted are values of a given spike train statistic (x-axis) for a range of neuron spike rates (y-axis) for three different models: a single-input rate model with high input signal variance (light red, wide diamonds), the same rate model with low input variance (dark red, thin diamonds), a stationary gamma renewal process with shape parameter 0.5 (blue, squares), and the BSRM with 6 ms refractory period (purple, circles). LV (**i**) and CV2 (**ii**) values of the BSRM overlap substantially with the high-variance input rate coding model (red shaded region) and stationary gamma renewal process (blue shaded region). Each line in (**A**) and (**B**) denotes the mean ISI distribution (autocorrelation) over ten trials, where each trial value was calculated from a 5000 second long spike train. Shaded regions give ± 1 standard deviation (over trials) and are negligibly thin. All data points in (**C**) were calculated from simulations of 4800 second long spike trains.

We first compared the ISI distribution and autocorrelation of a Poisson process^23^ and a gamma renewal process, both homogeneous and with absolute refractory periods, to those of our BSRM model. These models of spike generation regularly emit short ISIs, but without attributing a special function to them. Figure 6A, B shows that the BSRM model generates an ISI distribution and autocorrelation that are almost identical to those of the gamma process and even strikingly similar to the Poisson process. Since the BSRM is explicitly coding information in bursts but both other models are not, these results show that any metric based on the ISI distribution or the autocorrelation function will not always separate burst coding from rate coding cells.

Next, we tested whether previously used metrics of spike train irregularity, the Coefficient of Variation 2 (CV2; Fig. 6Ci)^33^ and the Local Variation (LV; Fig. 6Cii)^34^, were able to distinguish burst-multiplexing from non-burst-coding models. Both of these statistics take into account the relative order of ISIs in an effort to quantify the “local” variability in a spike train–that is, how similar adjacent ISIs are. This makes CV and LV2 both finer tools, and more robust to changes in firing rate than, for example, the coefficient of variation, or the ISI distribution comparison conducted in Fig. 6A. Intuitively, this might allow them to pick up on the added burst structure of the BSRM. To test this idea, we simulated the BSRM along with two non-bursting neuron models and checked whether the models could be parameterized to yield statistics indistinguishable from the BSRM. We found that both CV2 and LV of a Spike-Response Model (SRM^45^) neuron communicating a single input without a special meaning for bursts were distinct from the CV2 and LV of a BSRM only when the SRM received inputs with low variance. When the SRM received inputs with high variance, both the CV2 and LV were larger, covering almost the entire range of values produced by the BSRM. We found that CV2 $>1.2$ and LV2 $>1.4$ were not easily generated by the univariate SRM, although these values were generated by the BSRM at a low firing rate. We then asked if another non-burst coding neuron model would produce the CV2 and LV values in this range. We found that the stationary gamma renewal process—which approximates the firing statistics of the leaky integrate and fire model^46^—covered this range of values of CV2 and LV (blue lines in Fig. 6C). Together, the range of CV2 and LV values obtained from the BSRM while using bursts to communicate to streams of information is covered by realistic models of firing that do not utilize burst coding. Overall, we could not find a single-spike train metric that was able to reliably recognize burst coding at work.

## Discussion

This paper has, primarily, contributed two findings: (1) unimodal ISI distributions do not preclude burst coding in the form of burst multiplexing; (2) classic metrics for identifying bursty cells are unable to recognize when burst coding is being utilized. We close with a brief discussion of the mechanisms and implications of these findings, along with inspiration for future studies.

Given the inherent noisiness in spike trains and, thus, the burst multiplexing code, the driver and modulator channels might be better encoded and decoded in a probabilistic fashion. Specifically, we hypothesize that the graded synaptic efficacy employed in this study (Fig. 4) could be harnessed, in conjunction with Bayes’ rule, to encode the *probability* that a spike represents a burst or non-burst, thus communicating not only driver and modulator signals but also some notion of uncertainty about these signals.

Measures of local variation are unable to fully separate the BSRM from rate models because the input signal to a rate model can induce highly irregular spiking if it combines high power, for large fluctuations, with a slow fluctuation time scale. This results in short periods of supra-threshold input and fast, burst-like firing, followed by periods of semi-quiescence with long ISIs, when the signal fluctuations fall below threshold. Conversely, rapidly fluctuating input signals tend to result in less irregular spike trains, resulting in CV2 and LV values close to that of a homogeneous Poisson process (dark red lines in Fig. 6C). These two different input signal regimes result in very different SRM ISI distributions, with the rapidly-changing signal eliciting an ISI distribution akin to the Poisson process and the slowly fluctuating signal eliciting an ISI distribution similar to the Gamma renewal process. While CV2 and LV alone were unable to separate BSRM and SRM, augmenting these statistics with extra information, such as cell ISI distribution and firing rate, might reduce the possibility of misclassifying functionally bursty cells. For example, if the ISI distribution is similar to a Poisson process, making spike train irregularity due to input signal unlikely, but LV or CV2 are high, this might be suggestive of a functionally bursty spike train.

An important implication of our findings is that a larger class of cells could be utilizing bursts to code information than previous analyses have led us to believe. Furthermore, the robustness of burst multiplexing not only to the shape of ISI distributions but also to variants in synaptic decoding mechanisms suggests utility for the multiplexing code in diverse brain regions and cell networks. This has implications for the recently proposed theory that burst multiplexing is instrumental for the coordination of plasticity and for allowing biological networks to solve tasks that depend on hierarchical architectures^21^. We believe that our work provides grounds for heightened investigation of temporal codes in biological and artificial neural networks, for instance for the coordination of plasticity.

How, then, might one identify burst coding in the brain? Throughout this article, we have focused on features of the spike train alone, without any knowledge of either trial structure^47^, external stimuli^32,48^, behavioral state^17,31^ or simultaneously recorded neuronal activity^49^. These *accessory data methods* can and indeed have been used to detect if bursts add information that was absent from the firing rates^17,31,32,48^. In these studies, the shape of the ISI distribution was observed to change with external stimuli and state of attention in a manner that could not be accounted for by the associated changes in firing rates. To identify functionally bursty cells, methods such as those tested in the study, or related methods for burst detection^50–53^, could be adapted to take into account accessory data. In summary, just as the irregular firing patterns observed in the brain do not imply an unsteady neural code, a unimodal interspike interval distribution does not imply a rate code, as can be verified using accessory data.

## Methods

### Model

The network model was composed of a population of two-compartment burst-spike response model (BSRM) cells receiving identical synaptic inputs (Fig. 2B), and two cells post-synaptic to this encoding population. The two inputs to the encoding population controlled the event rate (driver input) and the burst probability (modulator input) of its neurons.

#### Input signals

**Table 1:** Power and timescale of the Ornstein-Uhlenbeck input signals.

Figure	$\sigma ^2_e$, $\sigma ^2_b$ (mV$^2$)	$\tau ^{(e)}$, $\tau ^{(b)}$ (ms)
Figures 3,4, 5 and 6 BSRM	1, 6	10, 20
Figure 6 SRM low power	1, N/A	10, N/A
Figure 6 SRM high power	10, N/A	100, N/A

Ornstein–Uhlenbeck (O–U) processes simulated via the exact method^54^ were used for the inputs, $\gamma _t^{(e)}$ and $\gamma _t^{(b)}$, where ‘e’ denotes event, or driver, and ‘b’ denotes burst, or modulator, signal. These were defined by

1 $$\begin{aligned} {\text{d}}\gamma _t^{(e)}&= - \frac{\gamma _t^{(e)}}{\tau _e}{\text{d}}t + \sqrt{\frac{2\sigma ^2_e}{\tau _e}} {\text{d}} W_t^{(e)} \end{aligned}$$

2 $$\begin{aligned} {\text{d}}\gamma _t^{(b)}&= - \frac{\gamma _t^{(b)}}{\tau _b}{\text{d}}t + \sqrt{\frac{2\sigma ^2_b}{\tau _b}} {\text{d}} W_t^{(b)} \end{aligned}$$

where ${\text{d}} W_t^{(x)}$ is the Wiener process (we note that the two Wiener processes, distinguished by different superscripts, are independent), $\tau _x$ is the time constant and $\sigma ^2_x$, $x \in \{e,b\}$, is its asymptotic variance. Higher variance and a larger time constant were used for the burst input as these were found to work better empirically. We expect the effect of the latter adjustment is because the variability inherent in intra-burst ISIs adds more noise to higher frequencies. See Table 1 for O–U process parameters.

#### Encoding cell model

**Table 2:** Model parameters for the encoding cells.

Name	Symbol (units)	Value
Cell resting potential	$v_0 \, ({\text{mV}})$	0
Burst threshold	$\theta _b \, ({\text{mV}})$	4.5
Burst scale factor	$\alpha _b \, ({\text{mV}})$	$\frac{1}{3}$
Burst ISI distribution scale	$\Gamma _1 \, ({\text{ms}})$	$\frac{20}{3}$
Burst ISI distribution shape	$\Gamma _2 \, ({\text{unitless}})$	1.5
Absolute refractory period	$\Delta _{\text{ref}} \, ({\text{ms}})$	2
Relative refractory time constant	$\tau _{\text{rel}} \, (\text{ms})$	Variable
Event threshold	$\theta _e \, ({\text{mV}})$	Variable
Event scale factor	$\alpha _e \, ({\text{mV}})$	2
Encoding population size	$N \, (\text{unitless})$	200

The BSRM is a modified version of the bursting rate model used in Naud and Sprekeler’s 2018 work^20^. The difference is that we explicitly modelled intra-burst spikes. Specifically, BSRM is a self-inhibiting marked point process defined by the double sequence $\{(B_n, {\mathscr {T}}_n) \mid n \in {\mathbb {N}}\}$ where ${\mathscr {T}}_n$ is a process with rate $\lambda _t$ and is constructed by alternating, as a function of the mark sequence, between sampling a modulated renewal process, for event spikes, and a renewal process, for intra-burst spikes. The rate is thus dependent on the mark process and is defined as follows

3 $$\begin{aligned} \lambda _t = \left\{ \begin{array}{cc} \rho (t) &{} \text {if} \quad \, B_{n} = 0 \\ \Gamma (t - {\mathscr {T}}_n) &{} \text {if} \quad \, B_{n}= 1 \\ 0 &{} \text{if} \qquad t - {\mathscr {T}}_n < \Delta _{\text{ref}} \\ \end{array} \right. \end{aligned}$$

where *n* is the index of the last spike and $B_n \sim \text{Bernoulli}\big (p({\mathscr {T}}_n)\big )$ if the $n-1^{th}$ spike is not the first spike in a burst in which case $B_n$ is fixed to 0. $\rho (t)$ is a function of the driver signal via

4 $$\begin{aligned} v^{(e)}(t)&= \eta (t-t^\prime ) + \gamma _t^{(e)} + v_0 \end{aligned}$$

5 $$\begin{aligned} \rho (t)&= f_{\text{link,e}}\big (v^{(e)}(t)\big ) \in [0, \infty ) \end{aligned}$$

and *p*(*t*) is a function of the modulator input via the equations

6 $$\begin{aligned} v^{(b)}(t)&= \gamma _t^{(b)} + v_0 \end{aligned}$$

7 $$\begin{aligned} p(t)&= f_{\text{link,b}}\big (v^{(b)}(t)\big ) \in [0, 1] \end{aligned}$$

Finally, $\Gamma (t)$ is the rate function for sampling an interval that is gamma distributed with scale parameter $\Gamma _1$ and shape parameter $\Gamma _2$ and $S_t = \sum _n \delta (t - {\mathscr {T}}_n)$ is the generated spike train, which is defined as a sum of Dirac delta functions over spike-time indices^45^. The marked process labels the first spike in a burst with a 1 and all other spikes with 0.

The above model describes the doublet BSRM that was used in most of the results. For the BSRM with *N* intra-burst spikes, used in Fig. 5, we simply sampled *N* ISIs after the first spike in the burst from the gamma distribution. Thus, the only difference from the doublet model is that there are now $N+1$ intra-burst spikes in a burst instead of just 2.

The following link functions were used to generate burst probability and event rate from the membrane potentials employed in the BSRM model.

8 $$\begin{aligned} f_{\text{link,b}}(v)&= \exp \bigg (\frac{v - \theta _{e}}{\alpha _{e}} \bigg ) \end{aligned}$$

9 $$\begin{aligned} f_{\text{link,e}}(v)&= \sigma \bigg (\frac{v - \theta _b}{\alpha _b} \bigg ) \end{aligned}$$

The exponential link function was chosen as it is commonly used in neural rate models^45^. The sigmoid link function, denoted by $\sigma$ above, was selected because it is the natural option for converting values on the positive real line to probabilities and has been used for burst modelling historically^20^. In both functions $\theta _x$ represents a threshold parameter and $\alpha _x$ determines the sensitivity of the threshold ($x \in \{e,b\}$).

An exponential function was used to model the relative refractory time period of the neurons, as is standard^45^, because the exponential decay reproduces the biological phenomenon. Using $t^\prime$ as the time of the last spike:

10 $$\begin{aligned} \eta (t - t^\prime ) = - \exp \bigg (- \frac{t - t^\prime }{\tau _{\text{rel}}} \bigg ) \Theta (t - t^\prime ), \end{aligned}$$

where $\Theta (x)$ is the Heaviside function with $\Theta (x) = 1$ if $x > 0$ and 0 elsewhere.

We will now outline our rationale for parameter choice in the BSRM model. The cell resting potential does not affect information transmitted and was set to zero for simplicity. The burst threshold and scale factor were chosen so that the fraction of total events that are bursts was approximately 0.2, as the burst fraction measured in layer 2/3 and layer 5 cortical cells is measured to be in the range 0.1–0.2^9^. The burst ISI scale parameter was chosen so that rate of spikes generated by a sequence of burst ISIs would be in the 100–200 Hz range observed in layer 5 cells^29^ and the shape parameter was selected to qualitatively produce the sharp, super-exponential peak observed in ISI distributions^30^. The 2 ms absolute refractory period is in line with the literature on cortical cells^55^ as well as cells in sub-cortical regions^56^.

Firing rate in cortical cells covers roughly two orders of magnitude, from around 1 Hz to tens of Hz^57^. Accordingly, the event threshold and scale factor were set to produce an event rate of approximately 10 Hz when $\tau _{\text{rel}}$ was set to 6 ms, which led to a value of 3.29 for the event threshold. For the uncorrected rate results (Fig. 3D) these initial event threshold and scale factor values remained fixed as $\tau _{\text{rel}}$ increased while for the rate corrected results the event threshold was decreased with increasing $\tau _{\text{rel}}$ to keep event rate fixed. Lastly, 200 BRSM cells were used for the encoding population as the goal was to explore a regime where spike generation (finite size effect) noise would be appreciable. See Table 2 for encoding cell parameters.

#### Decoding cells

**Table 3:** Model parameters for the decoding cells.

Name	Symbol (units)	Parameter value
Resting potential	$v_{syn} \, ({\text{mV}})$	1
Synaptic weight	${\bar{g}} \, ({\text{mV}})$	1
Synaptic rise time	$\tau _{\text{rise}} \, (\text{ms})$	3
Synaptic decay time	$\tau _{\text{decay}} \, (\text{ms})$	5
STP1 depression	$a, b \, \text{(none)}$	− 1, 0.5
STP1 facilitation	$a, b \, \text{(none)}$	1, − 0.5
STP2 depression	$a, b \, \text{(none)}$	40, − 20
STP2 facilitation	$a, b \, \text{(none)}$	Variable
STP ISI threshold	$\theta _{\text{w}} \, ({\text{ms}})$	Variable
STP smoothness	$\tau _{\sigma } \, ({\text{ms}})$	Variable

For the decoding cells, we adopted the model described in Ref.^41^, that defines STP weight functions as a composition of a linear filtering and nonlinear function. This can be formalized as

11 $$\begin{aligned} w(t) = f\big (a[ \kappa *S](t) + b \big ) \end{aligned}$$

where $f : {\mathbb {R}} \rightarrow [0, 1]$ is a nonlinear function, $\kappa (t)$ is a convolution kernel that we call the *weight function* because it encapsulates the frequency dependence of the synapse. The symbol ‘$*$’ denotes the convolution operation and *a* and *b* are parameters that are used to determine whether STP is facilitating or depressing. For sharp frequency dependence we set $f(x) = \Theta (x)$ and $\kappa (t) = \Theta (t) - \Theta (t - \theta _\text{w})$ and $\theta _\text{w}$ is the ISI threshold parameter, above which spikes are considered event-related and below which they are considered intra-burst. We will refer to this decoding model as STP1 and we note that it weighs each spike as a function solely of the ISI that came directly before it (renewal dynamics).

We also designed a STP rule with a smoother dependence of the weight function on ISI and a dependence on firing history beyond the just-preceding ISI (as used in Figs. 4, 5B). To achieve these desiderata, we chose $f(x) = \sigma (x)$ and $\kappa (t) = \Theta (t)\big [1 - \sigma \big (\frac{t - \theta _\text{w}}{\tau _\sigma }\big )\big ]$, where $\sigma$ denotes the sigmoid function and $\tau _\sigma$ is a parameter to control the smoothness of the function. We will refer to this model as STP2.

The above weight function definitions were inserted into the following equations which map spike trains of an encoding population of *N* cells to decoding-cell membrane potentials

12 $$\begin{aligned} A_t= & {} \frac{1}{N}\sum _{j=1}^N w^{(j)}(t)S^{(j)}_t \end{aligned}$$

13 $$\begin{aligned} u_t= & {} N[\kappa _{\text{syn}} *A](t) + v_{\text{syn}} \end{aligned}$$

In these equations, $A_t$ is the mean of weighted spike trains from the encoding population and $u_t$ is the synaptic response of the post-synaptic cell. In this way, the event rate is estimated in the membrane potential of a downstream cell employing depressing STP, $u^{(e)}$. To extract burst fraction, we must divide the burst rate by the event rate, an operation that has been shown to be implementable by neural machinery (i.e. divisive inhibition^20,37^). Rather than explicitly modelling such neural machinery, we simply took the ratio of the estimated burst and event rates $\frac{u^{(b)}}{u^{(e)}}$, where $u^{(b)}$ is the membrane potential of a neuron with short-term facilitation and estimating the afferent burst rate.

We found that information was better transmitted if a lag was introduced between $u^{(e)}$ and $u^{(b)}$ before taking their quotient. This makes sense given that the burst spike train lags behind the event spike train by the length of the intra-burst ISIs.

The synaptic filter $\kappa _{\text{syn}}$ was modelled as an exponential rise and exponential decay:

14 $$\begin{aligned} \kappa _{\text{syn}}(t) = {\bar{g}}\bigg [1 - \exp {\bigg (\frac{-t}{\tau _{\text{rise}}}\bigg )}\bigg ] \exp {\bigg (\frac{-t}{\tau _{\text{decay}}}\bigg )}. \end{aligned}$$

where ${\bar{g}}$ sets the scale of the synaptic conductivity.

The important aspect of the decoding cell parameterization was to arrive at quantities which were equivalent, in an information theoretic sense, to the synapses of decoding cells. For this reason their values were somewhat arbitrary, baring the following considerations. The resting membrane potential does not affect information transmission but, if set to zero, would result in an undefined estimate of burst fraction in a period of prolonged encoding population inactivity. The synaptic filter scale does not affect information and was set for simplicity. Because the synaptic filter itself is linear it will not affect information and was included simply for completeness.

To set the lag between burst and event signals in the decoded burst fraction for all experiments with doublet spikes, we performed a line search to maximize decoded information on lags between 0 and 15, with increments of 1ms. This was done for 3 BSRM cells with $\tau _{\text{rel}}$ values of 2 ms, 6 ms and 12 ms. Based on this we set the lag in all cases of doublet spikes to 9 ms.

To set the weight function thresholds we first assumed that there would be a single threshold, above which spikes would be considered event-related and below which they would be considered intra-burst spikes. This threshold, $\theta _\text{w}$, was selected uniquely for each cell model in the study by performing a line search on 5–35 ms (5–45 ms for Fig. 5B), in 1 ms increments, to maximize linear-decoding information.

The last parameters were those associated with STP rule 2. In Fig. 4*a* and *b*, the facilitation weight function parameters, were set to 40 and $-20$ respectively. In Fig. 5A these were set to $\frac{40}{N_{IBS}}$ and $-20$, where $N_{IBS}$ is the number of intra burst spikes modelled. In Fig. 4$\tau _\sigma$ was varied in 2 ms increments between 1 and 20 while in Fig. 5A it was set to 5 ms. See Table 3 for decoding cell parameters.

Finally, we note that, for STP1, Eq. (12) can be rewritten to match a previously used formalism^58^ for describing weighted spike trains

15 $$\begin{aligned} A_t = \frac{1}{N}\sum _{j=1}^N \sum _{i=1}^{N_{\text{spikes}, j}} \Theta \big (c(t - t_{i-1}^{(j)} - \theta _\text{w})\big )\delta (t - t_i^{(j)}) \end{aligned}$$

if, in Eq. (11), we set *f* to be the identity function, $a=1 \quad (-1)$, and $b=0 \quad (1)$ for facilitation (depression). Here $t_i^{(j)}$ denotes the *i*th spike of the *j*th neuron, $N_{spikes, j}$ denotes the number of spikes of the $j^{\text{th}}$ neuron, $c = 1$ if the synapse is depressing and $c=-1$ when the synapse is facilitating.

### Estimating linear-decoding information rate

**Table 4:** Hyper-parameters for the estimation of information.

Figure	*T* (s)	$N_{win}$ (s)
Figures 3 and 5Bii	1007.616	8.192
Figures 4 and 5Aii	503.808	4.096

To quantify information in our system we employ information rate, a tool commonly used in theoretical neuroscience^7,39^. Naively estimating information rate is difficult because it involves the non-parametric estimation of mutual information between high dimensional vectors, a task that requires prohibitively large data sets^59^. To make this estimation more tractable we rely on a method that makes use of the simple statistical structure of the Discrete Fourier Transform (DFT) of stationary Gaussian processes to estimate the linear-decoding information rate. The method used to estimate this rate throughout the results section is Stein’s method^40^.

Stein’s method is given by the following equation^40^:

16 $$\begin{aligned} {\mathbb {I}}_{lb}(X;Y) = -\int _{0}^{\frac{1}{2}} \log _2 \big (1 - \Phi _{XY}(f) \big ) df, \end{aligned}$$

where $\Phi _{XY}$ denotes the coherence between *X* and *Y*, which must be estimated from the data. This method requires the input signals to be stationary Gaussian processes, constraints which were satisfied by our use of O–U processes as stimuli signals.

To implement Stein’s method we estimated the power spectra of input and output processes and the cross spectra of the two, then used these to calculate the coherence. Estimation was performed with Welch’s method^60^, in Scipy, with a Hanning window. The parameters of this method are given in Table 4. Here *T* is simulation length and $N_{win}$ is window length. The overlap was chosen to be half the window length throughout. Simulations were run 5 times with different random seeds; all plots are the trial means.

In the course of this study we also considered the recently proposed correlation theory method^61^ as a means of estimating information rates, but decided against its use for reasons outlined in supplementary note A.

### On summing burst and event channel information rates

We used the sum of burst and event channel information rates, rather than the information rate between bivariate input and output signals. Because the bivariate information rate considers the full information between input and output vectors, it does not effectively quantify the extent that the inputs are “demixed” in the decoded outputs. For example, because mutual information is agnostic to invertible transformations, the bivariate method would assign equal information rates to a model whose decoded signals at a given time point are an invertible transformation of the input burst and event signals, at that time point, and a model that perfectly demixes the two inputs.

It was not immediately clear, however, whether the sum of mutual information rates would yield wrongly high rate estimates by counting information twice, as would occur if the input modulator and driver signals were precisely equal. This worry is easily resolved if one uses independent modulator and driver inputs, as in this study, which we prove below for completeness.

To prove that the sum of information rates does not count information twice, we show that the information rates for the burst and event channels is less than the information rate for the two channels combined when modulator and driver inputs are independent. We first make some definitions: the input and output stochastic processes are ${\mathscr {G}} = \{\Gamma _t\}_{t\ge 1}$ and ${\mathscr {U}} = \{U_t\}_{t\ge 1}$ respectively, where $\Gamma _t = [\Gamma _t^{(b)}, \Gamma _t^{(e)}]^\top$, $U_t = [U_t^{(b)}, U_t^{(e)}]^\top$ and the superscripts distinguish driver (*e* for event) and modulator (*b* for burst) *channels*. We define the $2 \times T$ array of *T* samples of the input process $\Gamma _{1:T} = [\Gamma _{1:T}^{(b)}, \Gamma _{1:T}^{(e)}]^\top$ and the analogous array, $U_{1:T}$, for the output process.

Using the definition of mutual information, and the independence of the input processes, we have

17 $$\begin{aligned}&I(\Gamma _{1:T};U_{1:T}) = H(\Gamma _{1:T}) - H(\Gamma _{1:T}|U_{1:T}) \nonumber \\&\quad = H\big (\Gamma _{1:T}^{(b)}\big ) + H\big (\Gamma _{1:T}^{(e)}\big ) \nonumber \\&\qquad - H\big (\Gamma _{1:T}^{(b)} | U_{1:T}\big ) - H\big (\Gamma _{1:T}^{(e)} | \Gamma _{1:T}^{(b)}, U_{1:T}\big ) \nonumber \\&\quad \ge H\big (\Gamma _{1:T}^{(b)}\big ) + H\big (\Gamma _{1:T}^{(e)}\big )\nonumber \\&\qquad - H\big (\Gamma _{1:T}^{(b)} | U_{1:T}^{(b)}\big ) - H\big (\Gamma _{1:T}^{(e)} |U_{1:T}^{(e)}\big ) \nonumber \\&\quad = I\big (\Gamma _{1:T}^{(b)};U_{1:T}^{(b)}\big ) + I\big (\Gamma _{1:T}^{(e)};U_{1:T}^{(e)}\big ) \end{aligned}$$

where we used the fact that entropy decreases (or is unchanged) by conditioning on another random variable^62^ to get the inequality.

This shows that the mutual information in a bivariate system with independent inputs is greater than the sum of the element-wise mutual informations. It remains to check that this extends to information rates.

We define the information rate between ${\mathscr {G}}$ and ${\mathscr {U}}$ as the difference of entropy rates, as in^63–65^:

18 $$\begin{aligned}&{\mathbb {I}}({\mathscr {G}};{\mathscr {U}}) := \lim _{T\rightarrow \infty }\frac{H(\Gamma _{1:T})}{T} - \lim _{T\rightarrow \infty }\frac{H(\Gamma _{1:T} \mid U_{1:T})}{T} \end{aligned}$$

Assuming both limits exist we thus have

19 $$\begin{aligned} {\mathbb {I}}({\mathscr {G}};{\mathscr {U}}) = \lim _{T\rightarrow \infty }\frac{I(\Gamma _{1:T};U_{1:T})}{T} \end{aligned}$$

By Eq. (17), we have

20 $$\begin{aligned} \frac{I(\Gamma _{1:T};U_{1:T})}{T} \ge \frac{I(\Gamma _{1:T}^{(b)}; U_{1:T}^{(b)})}{T} + \frac{I(\Gamma _{1:T}^{(e)}; U_{1:T}^{(e)})}{T} \end{aligned}$$

for all *T*. If the limits, with respect to *T*, of all three terms in Eq. (20) exist, taking the limit of both sides extends the result to information rates as desired.

### Metrics of spike train irregularity

CV2 was first suggested in Ref.^33^ and is defined as follows:

21 $$\begin{aligned} CV2 = \frac{2}{n-1}\sum _{i=1}^{n-1} \frac{|\text{ISI}_{i+1} - \text{ISI}_i|}{\text{ISI}_{i+1} + \text{ISI}_i} \end{aligned}$$

LV was developed in Ref.^34^ and is given by

22 $$\begin{aligned} LV = \frac{3}{n-1}\sum _{i=1}^{n-1} \frac{(\text{ISI}_{i+1} - \text{ISI}_i)^2}{(\text{ISI}_{i+1} + \text{ISI}_i)^2} \end{aligned}$$

In both equations we define $\{\text{ISI}_i\}_{i=1}^n$ as a sequence of ISIs calculated from a single spike train, so that $\text{ISI}_{i+1}$ follows directly after $\text{ISI}_i$.

### Recorded data

The dataset used in Fig. 1 consists of 1266 spike trains recorded from multiple regions of the mouse brain using neuropixel probes, and has previously been published^8^. We used Hartigan’s dip test ($p \le 0.05$)^66^ to separate unimodal from multi-modal ISI distributions.

## Supplementary Information

Supplementary Information.

## Data Availability

https://github.com/nauralcodinglab/zeke_msc.
