# Supplementary material for: Neural burst codes disguised as rate codes
Source: Sci Rep. 2021 Aug 5;11:15910. doi: 10.1038/s41598-021-95037-z (PMC8342467; doi:10.1038/s41598-021-95037-z)
Supplement: Supplementary file 1 — Supplementary Information. [file 41598_2021_95037_MOESM1_ESM.pdf]

A Supplementary Information: Correlation theory

Correlation theory[1] is a recently proposed method to estimate the information rate of neural spike trains. We investigated correlation theory as a second method to evaluate burst coding in neural populations but, in doing so, found that this method is not generally an estimate of non-linearly encoded information. We outline these findings here.

In the original paper, correlation theory is presented as a method for calculating the information rate between two time series, which we will denote throughout this section by X and Y. To make things more tangible, X will be considered to be the input to a system, e.g. a synaptic stimulus, and Y the output, e.g. a neural spike train. We will further define $Y_{|x}$ to be the output time series conditioned on a sample path, x, of the input.

To prove that correlation theory calculates the full information rate between X and Y, Dettner et al. (2016)[1] used the result, due to David Brillinger, that if a T-length sample from a given time series, represented as vector $Y^{(T)}$, is mean-zero, finite memory and stationary, it has Fourier components that are asymptotically[2] distributed as follows

$$\hat{Y}_f^{(T)} \xrightarrow{d} \mathscr{N}\big(0, \mathscr{P}_{YY}(f)\big) \quad \text{as } T \to \infty \tag{1}$$

where $\hat{x}_f$ denotes the discrete Fourier transform of vector x at frequency f, $\mathscr{P}_{YY}$ the 2 x 2 diagonal matrix whose non-zero elements are the power spectrum of Y, and $\mathscr{N}$ denotes the bivariate normal distribution; we note that this is a bivariate value because the discrete Fourier transform is generally a complex number, with a real and imaginary part.

Critically, it is further assumed by correlation theory that if one can show empirically that the discrete Fourier transforms of $Y_{|x}$ are also asymptotically normal, then the information rate is given by the correlation theory equation

$$\mathbb{I}_{\text{corr}}(X;Y) = \int_0^{\frac{1}{2}} E_x\left[\log_2\left(\frac{\sigma^2(f)}{\sigma_{\hat{x}}^2(f)}\right)\right] df \tag{2}$$

where the expected value is over sample paths of X, and $\sigma^2(f)$ and $\sigma_{\hat{x}}^2(f)$ are the variance of the real and imaginary parts of $\hat{Y}$ and variance of the real and imaginary parts of $\hat{Y}_{|x}$, respectively. Note that one must compare the mean-subtracted time series because otherwise the moments of the Fourier transforms at $f = 0$ will not be defined.

If one can show that there exist time series X and Y where $\hat{Y}_{|x}$ is asymptotically normal such that the above expression fails to calculate the information rate of X and Y then correlation theory is incorrect for stationary, finite memory time series in general. We will show that such time series exist.

Let $X_t \sim \mathscr{N}(0,1)$ and $N_t \sim \chi^2(1)$, for all t, where $\mathscr{N}$ denotes the normal distribution and $\chi^2(n)$ denotes the chi-square distribution with n degrees of freedom. Next, define $Y_t = X_t^2 + N_t \,\forall\, t$. X_t and Y_t are thus trivially jointly stationary and finite memory. Now assume that each of X_t^2, N_t and Y_t are shifted so that their means are zero, allowing for Fourier transforms that are defined even at the zero frequency. Their information rate is given thus:

$$\mathbb{I}(X,Y) = \lim_{T\to\infty} \frac{I([X_1,\ldots,X_T];[Y_1,\ldots,Y_T])}{T} \tag{3}$$

$$= \lim_{T\to\infty} \frac{T}{T} I(X_1;Y_1) \tag{4}$$

$$= \frac{1}{2} - \log_2(\sqrt{\pi}) - \frac{1}{2}\psi(\frac{1}{2}) \tag{5}$$

where we have used the expression for the entropy of a chi-square distribution in units of bits and the fact that shifting the location of a distribution does not affect its variance or entropy.

Now we will compute the value of the correlation theory expression. By Brillinger's theorem the Fourier transforms of the output time series are described by equation 1. This means that $\sigma^2(f) = \mathscr{P}_Y(f)$. This is the power spectrum of a stationary time series so it is equal to the Fourier transform of the autocorrelation of Y. Because Y are iid samples the autocorrelation is zero everywhere except at $\tau = 0$, where it is given by the correlation function for a chi-square random variable, Z, with 2 degrees of freedom; thus $\mathscr{P}_Y(f) = E(Z^2) = 6 \,\forall\, f$.

By linearity of the Fourier transform

$$\hat{Y}_{|x,f} = \widehat{(x^2)}_f + \hat{N}_f \tag{6}$$

Thus, the randomness of $\hat{Y}_{|x,f}$ is fully determined by $\hat{N}_f$. This means that $\hat{Y}_{|x,f}$ is normal by the asymptotic normality of the noise process. Also by Brillinger's proof, the variance of the real and imaginary components of $\hat{Y}_{|x,f}$ is equal to the power

spectrum of the noise process. Because the noise process is stationary this is simply the Fourier transform of the autocorrelation function, which is zero everywhere except for zero time lag, by iid sampling, where it is equal to the correlation of a chi square random variable with one degree of freedom; thus $V(\hat{Y}_{|x,f}) = 3\,\forall f$. Taken together with $\mathscr{P}_Y(f)$ and equation 2, we get

$$\mathbb{I}_{\text{corr}}(X;Y) = \int_0^{\frac{1}{2}} E_x\left[\log_2\left(\frac{6}{3}\right)\right] df \tag{7}$$

$$= \int_0^{\frac{1}{2}} \log_2(2)\, df = \frac{1}{2} \tag{8}$$

completing our counter argument. We expect that the issue is that correlation theory assumes convergence in entropy will follow from convergence in distribution, which is not always true[3].

We have shown here that correlation theory does not estimate information rate for stationary, finite memory time series in general. For specific time series, e.g. neural spike trains, the required entropy convergence may indeed occur, but, to our knowledge, this remains to be proven. Regardless, as shown in[1], correlation theory is equal to Stein's method[4] in the case of a linear transfer function and is thus an estimator for information in linear regimes.

B Supplementary Information: Figures

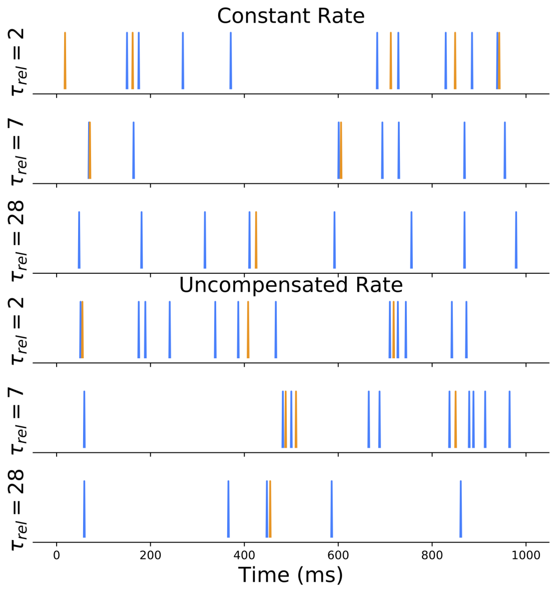

Figure S 1. Sample Spiketrains from BSRM. Spike trains with same statistics as in Fig.3A of the main paper for the constant rate condition (top three rows) and uncompensated rate condition (bottom three rows). X-axis is time (in milliseconds). The lines denote spike times, with orange lines for bursts and blue for events. Y-label gives the value of τ_{rel}, the refractory period time constant.

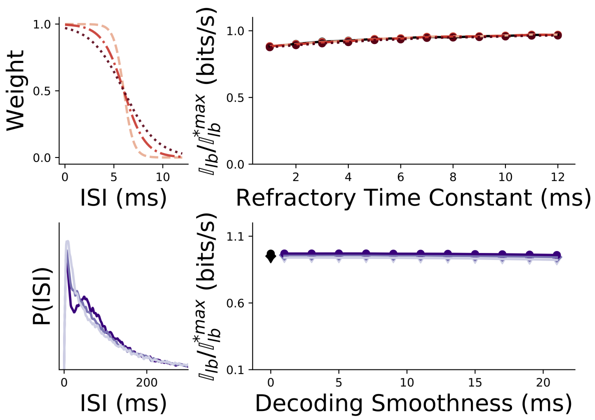

Figure S 2. Properties of Dynamic Synapses Influence Information Transmission - Driver Channel. Exactly as in Fig.4.C,D of the main paper except plotting data for the driver channel instead of the modulator.

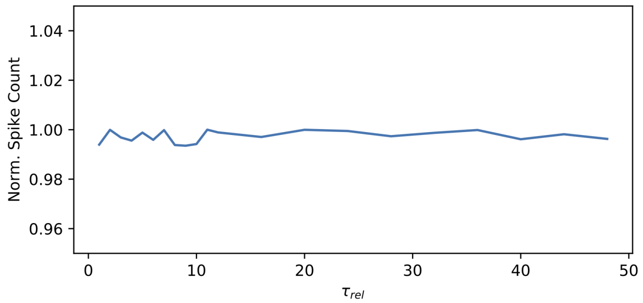

Figure S 3. Graphical demonstration that rate correction in Fig.3 of the main paper was correctly implemented. Y -axis is the total spike counts per ISI distribution, normalized by the maximum of these over parameter values. X-axis is parameter value.

References

1. Dettner, A., Münzberg, S. & Tchumatchenko, T. Temporal pairwise spike correlations fully capture single-neuron information. *Nat. communications* **7**, 13805 (2016).
2. Brillinger, D. R. *Time series: data analysis and theory* (SIAM, 2001).
3. Piera, F. J. & Parada, P. On convergence properties of shannon entropy. *Probl. Inf. Transm.* **45**, 75–94 (2009).
4. Stein, R. B., French, A. S. & Holden, A. V. The frequency response, coherence, and information capacity of two neuronal models. *Biophys. journal* **12**, 295–322 (1972).
